# How good is the evidence that cellular senescence causes skin ageing?

**DOI:** 10.1016/j.arr.2021.101456

**Published:** 2021-11

**Authors:** Evon Low, Ghazaleh Alimohammadiha, Lucy A. Smith, Lydia F. Costello, Stefan A. Przyborski, Thomas von Zglinicki, Satomi Miwa

**Affiliations:** a Ageing Biology Laboratories, Newcastle University Biosciences Institute, Newcastle University, Newcastle upon Tyne NE4 5PL, UK; b Department of Biosciences, Durham University, South Road, Durham DH1 3LE, UK

**Keywords:** skin, senescence, ageing, wound healing

## Abstract

Skin is the largest organ of the body with important protective functions, which become compromised with time due to both intrinsic and extrinsic ageing processes. Cellular senescence is the primary ageing process at cell level, associated with loss of proliferative capacity, mitochondrial dysfunction and significantly altered patterns of expression and secretion of bioactive molecules. Intervention experiments have proven cell senescence as a relevant cause of ageing in many organs. In case of skin, accumulation of senescence in all major compartments with ageing is well documented and might be responsible for most, if not all, the molecular changes observed during ageing. Incorporation of senescent cells into in-vitro skin models (specifically 3D full thickness models) recapitulates changes typically associated with skin ageing. However, crucial evidence is still missing. A beneficial effect of senescent cell ablation on skin ageing has so far only been shown following rather unspecific interventions or in transgenic mouse models. We conclude that evidence for cellular senescence as a relevant cause of intrinsic skin ageing is highly suggestive but not yet completely conclusive.

## Highlights

• Senescent cells accumulate in all major compartments of the skin during ageing.
• Senescent cells have the potential to drive typical skin ageing phenotypes.
• Impact of specific senescence modifications on skin ageing is not fully established.
• Role of senescence for chronic wound healing needs experimental clarification.

## 1 Ageing of the skin

**Fig. 1:**
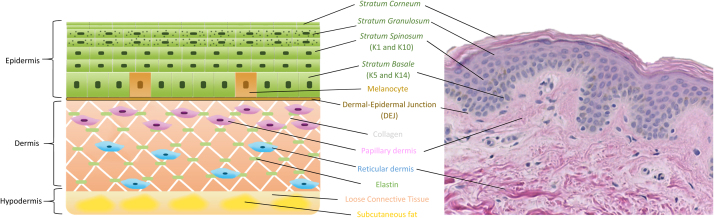
Human skin structure. The epidermal layer is separated from the underlying dermis by a basement membrane. The epidermis is mainly composed of keratinocytes. During cell differentiation, cells become flatter with thickening of cell membranes as they are pushed towards the *stratum corneum*. The stages of differentiation are characterized by expression of specific keratins as listed above. In the *stratum granulosum*, smaller dark purple dots in the cells represent lamellar granulosum that serve as water barrier. Melanocytes locate within the *stratum basale* of the epidermis. They provide skin color and protect skin from UV damage. The dermis is a fibrous layer rich in collagen and elastin that are produced by fibroblasts. Hypodermis is the deepest layer of the skin that is made up of loose connective tissues and fat. For interpretation of the references to color in this figure legend, the reader is referred to the web version of this article.

Skin is a complex organ that comprises several compartments with different functions. It is the largest organ covering the human body, with a surface area of approximately 1.5–2 m^2^, and has direct contact with the external environment. The skin consists of three layers, namely the epidermis, dermis, and hypodermis (Fig. 1). The epidermis is the outermost layer that has a protective function to prevent penetration of pathogens and regulate water loss from the body. The dermis contains a dense extracellular matrix network that provides the skin with mechanical strength and elastic recoil, and the subcutaneous fat within the hypodermis provides insulation.

The epidermis has a stratified, squamous organization with a multitude of cell types including mechanosensory Merkel cells, antigen-presenting Langerhans cells, pigmented melanocytes, and keratinocytes, which are the most abundant cell type (Brenner and Hearing, 2008). Epidermal stem cells within the *stratum basale* give rise to progenitor cells, which undergo programmed, sequential differentiation to form the suprabasal *stratum spinosum*, *stratum granulosum* and terminally differentiated *stratum corneum*.

The dermis has a complex architecture, maintained by dynamic reciprocity between the dermal-resident cells and a composite network of extracellular matrix. The dermis contains two morphologically distinct layers known as the papillary and reticular dermis, which differ in structure and composition. The papillary dermis contains fine and sparsely arranged collagen, oxytalan and elaunin fibers, with a perpendicular orientation to the dermal-epidermal junction. The reticular dermis contains a dense network of collagen and elastic fibers, embedded within a non-fibrous ground substance of glycoproteins, proteoglycans and glycosaminoglycans. Fibroblasts in the papillary and reticular compartments differ by morphology, growth potential, secretion of cytokines and matrix-modifying enzymes (Mine et al., 2009), lineage marker expression and differentiation potential (Korosec et al., 2019) and their ability to support keratinocyte differentiation (Janson et al., 2017, Lämmermann et al., 2018, Mine et al., 2008).

The hypodermis resides beneath the dermis, and it connects the skin to the underlying fascia. The hypodermal adipose tissue is composed of lobules of adipocytes separated by connective tissue septae, which provide the skin with insulation and storage (Khavkin and Ellis, 2011).

The principal function of skin is to act as a barrier, which protects against potential exterior insults such as pathogens, chemicals and physical stressors and helps to maintain the body’s interior homeostatic balance. The responsibility for providing the physical barrier function rests mainly on the keratinocytes of the epidermis, with the *stratum corneum* contributing significantly to barrier properties (Choi, 2019). The skin is not entirely impermeable; water loss to the external environment, also known as transepidermal water loss (TEWL), contributes to water loss from the body, and is also used as one of a range of methods to functionally assess the skin’s barrier function. Other surrogate markers that are accepted as indicative of barrier function include *stratum corneum* hydration, sebum levels and pH (Luebberding et al., 2013). More indirect techniques, e.g., tape stripping may also be used to analyze the lipid composition of the *stratum corneum*, either through mass spectroscopy of samples or via techniques such as corneosurfametry, where the lipid composition of tape stripped, samples is analyzed using histological stains and colorimetry. These techniques can be adapted to measure the impact of interventions on (aged) skin and in 3D organotypic models (Alexander et al., 2018, Srinivasan et al., 2015).

**Fig. 2:**
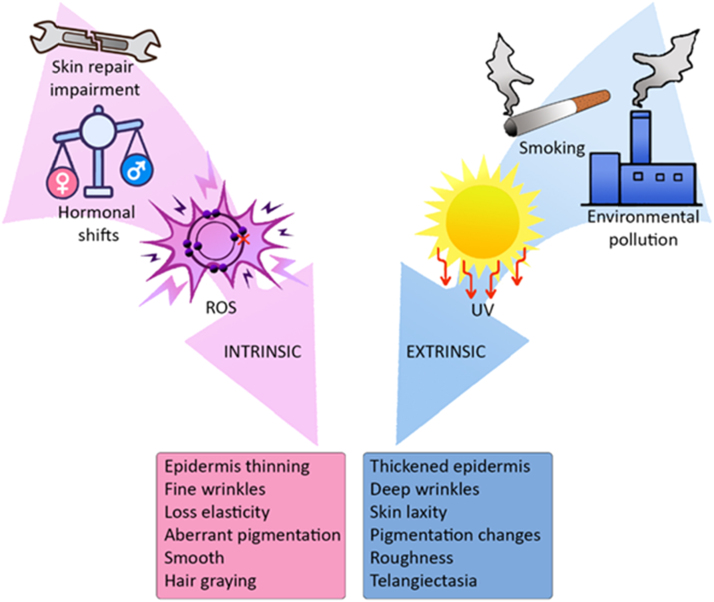
Skin ageing is driven by both intrinsic (e.g., ROS, skin repair impairment, hormones) and extrinsic (e.g., smoking, UV exposure, environmental pollution) factors. Ageing phenotypes caused by intrinsic factors are partially different from those driven by extrinsic ageing. Characteristic signs of intrinsic ageing include epidermal thinning, fine wrinkles, loss of elasticity, aberrant pigmentation, hair graying and smooth appearance. Factors such as UV exposure, cigarette smoke and environmental pollutants drive extrinsic skin ageing. Extrinsically aged skin appears thick and rough, with coarse wrinkles, skin laxity, pigmentation changes and visible appearance of blood vessels under the skin surface (telangiectasia).

Skin ageing is a process associated with both intrinsic and extrinsic factors that leads to a progressive loss of structural integrity and physiological function (Friedman, 2005), ( Fig. 2).

Extrinsic skin ageing is defined as the cumulative effects of lifestyle and environmental factors including ultraviolet radiation (UV), smoking and environmental pollution. More comprehensively, (Krutmann et al., 2017) introduced the concept of the skin ageing exposome and defined the environmental factors which are part of it as belonging to the following major categories: (i) sun radiations: ultraviolet radiation, visible light and infrared radiation, (ii) air pollution, (iii) tobacco smoke, (iv) nutrition, (v) a number of less well studied, miscellaneous factors, as well as (vi) cosmetic products.

Phenotypically, extrinsic skin ageing is characterized by deep wrinkles, skin laxity, roughness, pigmentation changes and visible appearance of blood vessels under the skin surface, known as telangiectasia (Wlaschek et al., 2001). Unlike intrinsically aged skin, extrinsic skin ageing (UV-irradiated) demonstrates a thickened epidermis, especially in the *stratum corneum (*Kligman, 1989*)* due to corneocyte desmosomes failing to degrade effectively. Following UV irradiation, involucrin was increased in the *stratum corneum*, whilst β1-integrin was greatly reduced in basal cells, indicating that UV causes the impairment of keratinocyte differentiation (Makrantonaki and Zouboulis, 2007). In young skin, elastin and collagen fibers are tightly packed and highly organized, but in extrinsic skin ageing the organization of collagen bundles is altered (Quan and Fisher, 2015, Yasui et al., 2013) associated with collagen deficiency. Specifically, collagen I is greatly diminished resulting in an increased ratio of type III to type I collagen. The loss of functional melanocytes and alterations in the interactions between melanocytes and keratinocytes leads to protective barrier dysfunction against UV radiation (Brenner and Hearing, 2008). Furthermore, in response to extrinsic ageing factors such as exposure to UV radiation, alterations in TEWL have been observed as an acute stress response (Choi, 2019).

The induction of photoaging together with multiple markers of cell senescence by UVB in vivo has been demonstrated using p16INK4A reporter mice (Sorrentino et al., 2014) and there is extensive literature on the induction of senescence and skin aging by multiple components of the skin exposome including the UV modalities UVA and UVB (Krutmann et al., 2021).

Intrinsic skin ageing is described as a natural consequence of physiological changes over time such as the generation of reactive oxygen species (ROS), hormonal shifts, impairment to repair skin damage (Farage et al., 2008) and, possibly, cell senescence. It is characterized by visible signs of epidermis thinning, fine wrinkles, loss of elasticity, aberrant pigmentation, smoothness, and hair graying. Skin thinning is the most remarkable change in intrinsic skin ageing (Waller and Maibach, 2005). The epidermis retains its multi-layered, keratinized structure with age, however, decreased epidermal thickness, reduced epidermal turnover and strata-specific alterations have been well documented (Table 1). Dermal ageing is associated with chronic atrophy, attributed to the degradation of the extracellular matrix networks, which causes reduced elasticity, decreased extensibility and increased laxity of the skin (Table 1). The distance between dermo-epidermal junction decreases and leads to insufficient nutrition supply to the epidermis ultimately reducing basal cell proliferation. In sun-protected areas, the aged dermis contains fewer mast cells, fewer fibroblasts (especially papillary fibroblasts) and less collagen, as well as elastic fibers as compared to sun-protected young dermis (Kohl et al., 2011). It was stated that the thinning of superficial dermis is due to gradual lysis of connective tissues, while the increase in density is caused by the thinnest fibers being degraded first, leaving thicker collagen fibers in the deep dermis. This may also explain why the resistance of the skin weakens at the surface (Bonta et al., 2013). The loss of collagen content is associated with upregulation of collagen degrading enzymes, i.e., matrix metalloproteinases (MMPs) (Rittié and Fisher, 2002) along with a decrease in collagen synthesis. The age-related loss of subcutaneous fat from the hypodermis contributes to the wrinkling and sagging of ageing skin (Ezure and Amano, 2010).

**Table 1:** Structural changes of human skin during intrinsic ageing of sun-protected skin.

Layer	Sublayers	Age-related changes	Expected physiological and pathological consequences
Epidermis	General	Decreased epidermal thickness	May lead to changes in barrier function, such as increased water loss and susceptibility to infections (Rorteau et al., 2020)
	*Stratum corneum*	Altered lipid composition	Changes in barrier function and skin dryness (Starr et al., 2016)
	*Stratum granulosum*	No significant difference in keratinocyte size, nuclear size or nuclear: cytoplasmic ratio (Liao et al., 2013)	–
	*Stratum spinosum*	Apoptosis of keratinocytes (Gilhar et al., 2004)	May reflect reduced proliferative capacity, leading to reduced epidermal thickness (Rübe et al., 2021)
	*Stratum basale*	Reduced proliferative capacity of basal keratinocytes (Rübe et al., 2021) Reduced height of basal keratinocytes (Sauermann et al., 2002) Increased size of basal keratinocytes and nuclei (Liao et al., 2013) Decreased number of melanocytes (Gilchrest et al., 1979)	Changes in the balance between differentiation and proliferation of basal keratinocytes, resulting in reduced maintenance of stratified layers (Charruyer et al., 2021, Rorteau et al., 2020) and an overall reduction in epidermal thickness (Gilhar et al., 2004). Irregular skin pigmentation, both hyperpigmentation and hypopigmentation (Lee et al., 2021a, Lee et al., 2021b, Lee, 2021)
Dermo-epidermal junction	–	Reduced height of dermal papilla (Sauermann et al., 2002) Reduced height of rete ridges and flattening of dermo-epidermal junction (Newton et al., 2017)	May lead to changes in skin barrier function (increased water loss and infection susceptibility) (Rorteau et al., 2020), as well as reduced resistance to shearing (Langton et al., 2019)
Dermis	Papillary	Decreased quantity and thickness of collagen bundles (Marcos-Garcés et al., 2014) Altered orientation of collagen fibers in the reticular dermis (Marcos-Garcés et al., 2014) Decreased numbers of fibroblasts with papillary morphology (Mine et al., 2009)	Impact on biomechanics of skin, specifically elasticity and stiffness (Langton et al., 2019) which may lead to increased instability and wrinkling (Kruglikov and Scherer, 2018)
	Reticular	Collapsed morphology of fibroblasts (Fisher et al., 2008) Altered composition of fibroblast secretome with an upregulation of proinflammatory cytokines, pro-oxidant components and matrix degrading factors (Waldera Lupa et al., 2015) Chronic degradation of the collagen and elastin networks by matrix metalloproteinases (Ashworth et al., 1999) Reduction in tissue inhibitor of metalloproteinases (TIMPs) Decreased quantity of interstitial collagen (Varani et al., 2006) 40% reduction in the ground substance (Li et al., 2013)	Imbalance between ECM synthesis and degradation, leading to changes in architecture and loss of dermal volume (Rorteau et al., 2020) Impact on skin biomechanics - loss of skin elasticity and increased dermal stiffness (Langton et al., 2019, Strnadova et al., 2019) Increased risk of lacerations, delayed wound healing and increased risk of infections and bleeding (Strnadova et al., 2019, Wollina et al., 2019)
Hypodermis	–	Relative loss of subcutaneous fat (Ezure and Amano, 2010)	Reduced protection from infection and trauma and potential metabolic disturbances (Tchkonia et al., 2010)

Epidermal thinning and flattening of the dermo-epidermal junction can result in increased skin fragility and reduced resistance to shearing forces. These lead to increased potential for barrier disruption and delayed barrier repair, although *stratum corneum* thickness and TEWL may be maintained during chronological ageing (Rittié and Fisher, 2015). Changes in skin pH occur during aging, impacting on the activity of enzymes involved in *stratum corneum* barrier formation (Schmid-Wendtner and Korting, 2006, Wang et al., 2020).

A range of diverse molecular mechanisms has been shown to be associated with skin ageing. Some of the major mechanisms are:

**Oxidative stress** is thought to be an important contributing factor in both intrinsic and, to an even larger extent, extrinsic skin ageing and dermal damage (Kammeyer and Luiten, 2015, Rittié and Fisher, 2002). Reactive oxygen species (ROS) are mainly generated by mitochondria and can be protective against skin infection by activating hypoxia-inducible factor 1-alpha (HIF1α) and recruiting immune cells (Hamanaka and Chandel, 2009). However, the accumulation of excessive ROS can cause damage to macromolecules (lipids, DNA and proteins) leading to cellular dysfunction. For instance, accumulation of cellular lipofuscin, containing oxidized lipids and cross-linked proteins, has been detected in fibroblasts and keratinocytes during ageing (Katz and Robison, 2002). Generation of macromolecular damage interferes both directly and indirectly with the major proteostatic systems of the cell, the **Ubiquitin-proteasome system** and the **autophagosome/lysosome** system (Korovila et al., 2017). Elevated ROS production activates the mitogen-activated protein kinase (MAPK) pathway, which consequently stimulates the production of activator protein 1 (AP-1). AP-1 transcriptionally regulates matrix metalloproteinases (MMPs) which determine composition and amount of the extracellular matrix (ECM) in the dermis. MMP-1 cleaves collagen, which is further degraded by MMP-3 and MMP-9. On the other hand, tissue inhibitors of metalloproteinases (TIMPs) are downregulated during the ageing process (Fisher and Voorhees, 1998). UV radiation can also stimulate MMP expression, perhaps through the DNA damage response (DDR) pathway leading to the activation of MAPK and transforming growth factor-beta 1 (TGF-β1). Among other effects, TGF-β1 regulates the production and degradation of collagen in the ECM, thus affecting the structure and integrity of dermal fibroblasts (Quan et al., 2010).

**Mitochondrial DNA (mtDNA)** and nuclear DNA damage and epigenetic changes. DNA damage can occur by endogenous mitochondrial ROS production. Mitochondrial DNA mutations, including deletions and point mutations of mtDNA, accumulate in skin during ageing and following UV irradiation. mtDNA damage can exacerbate mitochondrial ROS production, it can also induce premature aging in model systems (Kujoth et al., 2005, Trifunovic et al., 2004) including wrinkle formation and hair loss (Singh et al., 2018).

Shifts in histone chromatin marks, including markedly reduced levels of H4K16ac, absence of high H4K20me1 staining and increased cell-to-cell variability in total histone H3 and H4 content, are characteristic for ageing in the basal keratinocytes (Dube et al., 2021) together with decreased expression of multiple histone deacetylases (Lee, 2021, Lee et al., 2021b, Lee et al., 2021a). DNA methylation changes characteristically with age in human keratinocytes and fibroblasts, providing a basis for a skin cell-based methylation clock and, possibly, for the validation of anti-ageing interventions in skin (Boroni et al., 2020, Horvath et al., 2018).

Skin ageing may also be driven by damage to nuclear DNA, especially to telomeres (Aguado et al., 2019, Victorelli and Passos, 2020). **Telomeres** not only shorten gradually with age due to the end replication problem but also accumulate damage that causes premature shortening (von Zglinicki, 2002) and/or induction of a persistent DNA damage response at telomeres (Herbig et al., 2006, Hewitt et al., 2012) which is an efficient trigger of cell senescence. In mice, telomere shortening impaired the function of epidermal stem cells (Flores et al., 2005). In humans, basal cells in UV exposed skin regions have been found to have shorter telomeres in comparison to sun-protected regions (Ikeda et al., 2014), which may be explained by the fact that UV radiation specifically targets telomeric GGG triplets (Oikawa et al., 2001). In sun-protected human epidermis, little or no telomere loss with ageing was found (Krunic et al., 2009, Victorelli et al., 2019). This might be due to the fact that keratinocytes - at least in the basal stem cells - express active telomerase (Härle-Bachor and Boukamp, 1996, Krunic et al., 2009). However, the number of skin cells displaying a telomeric DDR independently of telomere length (indicated by telomere-associated DNA damage foci, TAF) increased significantly with age in the skin of baboons (Herbig et al., 2006) and in melanocytes and keratinocytes in situ in human skin (Victorelli et al., 2019).

**Cytokine production** is altered during skin ageing leading to imbalances of various proinflammatory components such as interleukins and tumor necrosis factor alpha (TNF-α) that stimulate upregulation of metalloproteinases including MMP-9. Interleukins (IL)-1 and IL-18 accumulate with age and contribute to skin inflammation and IL-1, IL-6, IL-8, IL-10, IL-15, and TNF- α increase after UV exposure in keratinocytes (Strickland et al., 1997) and dermal fibroblasts (de Kossodo et al., 1995). Fibroblasts from intrinsically aged skin increase production of cysteine-rich protein 61 (CCN1), which causes alteration of collagen homeostasis leading to skin dysfunction (Waldera Lupa et al., 2015).

As this incomplete overview of molecular mechanisms shows, skin aging is a multi-causal process with loss of tissue and cell homeostasis, loss of proteostasis, increased protein oxidation, reduced immune capacities for senescence clearance, impaired DNA repair competence and others. Thus, it can be doubted whether cellular senescence is decisive for all this changes. It is, however, intriguing that most, if not all, of the above indicated molecular mechanisms associated with skin ageing are similarly known in cellular senescence. For instance, cell senescence is typically driven by telomere dysfunction and ensuing DNA damage response (d'Adda di Fagagna et al., 2003), stabilized by mitochondrial dysfunction and production of high levels of ROS (Correia-Melo et al., 2016, Passos et al., 2010) as well as by cytokine (SASP) release (Acosta et al., 2008, Campisi et al., 2019, Coppé et al., 2010) and accompanied by major epigenetic reprogramming (Shah et al., 2013) and autophagy/mitophagy dysfunction (Korolchuk et al., 2017) (for review, see Gorgoulis et al., 2019, von Zglinicki, 2021). This raises the possibility that cell senescence might be an underlying core mechanism of skin ageing.

In fact, cell senescence, especially senescence of dermal fibroblasts, has been postulated as a driver of skin ageing in many recent reviews (Campisi et al., 2019, Demaria et al., 2015, Fitsiou et al., 2021, Ho and Dreesen, 2021, Lee, 2021, Lee et al., 2021a, Lee et al., 2021b, Toutfaire et al., 2017, Velarde and Demaria, 2016, Wang and Dreesen, 2018, Wlaschek et al., 2021). There is already good evidence for a causal role of cell senescence in photoaging of the skin, which has been covered in multiple recent reviews (Cavinato and Jansen-Dürr, 2017, Fitsiou et al., 2021, Toutfaire et al., 2017, Velarde and Demaria, 2016). However, a driving role of cell senescence in intrinsic ageing of photo-protected skin is less well evidenced, and this will the primary focus of the reminder of this review.

## 2 Cellular senescence

### 2.1 What is cellular senescence?

Replicative senescence was first established in 1961 by Hayflick and Moorhead as the irreversible loss of proliferative capacity after a reproducible number of population doublings in culture (Hayflick and Moorhead, 1961). Later, replicative senescence was linked to telomere attrition as telomeres in somatic human cells shorten with each cell division (Harley et al., 1990). It was demonstrated that telomerase could protect telomeres from shortening, thus preventing replicative senescence (Bodnar et al., 1998). Progressive telomere loss results in chromosome ends recognized as double strand breaks (DSB), which provokes the activation of a DNA damage response (DDR) that, if it persists, causes senescence (d'Adda di Fagagna et al., 2003). Telomeres are not very accessible to the DNA repair machinery (Doksani and de Lange, 2014, Richter et al., 2007), resulting in persistent DNA damage foci that are potent inducers of senescence or apoptosis (Fumagalli et al., 2012, Hewitt et al., 2012).

In vivo, replicative exhaustion is just one inducer of senescence and other stresses, including DNA-damaging ROS, radiation or oncogene expression can be much more prominent. While telomere uncapping remains a general characteristic of senescent cells in the vast majority of stress response modes, a wide variety can be observed in senescent phenotypes and differences in the inducer mechanism is one determinant of this variety (Tigges et al., 2014, Waldera Lupa et al., 2015).

Multiple biomarkers are in use to detect the presence of senescent cells in vivo: A long-established marker is the expression of lysosomal β-galactosidase activity at sub-optimal pH (Senescence-Associated β-Galactosidase, SA-β-Gal), which increased with age in human skin (Dimri et al., 1995). Many biomarkers of senescence are based on the upregulation of cell cycle inhibitors including p16INK4a, p21WAF1/CIP1, p53, ARF, p15 and p27 and downregulation of proliferation-associated proteins (Beauséjour et al., 2003). Additional markers are loss of LaminB1, a structural component of the nuclear lamina (Dreesen et al., 2013, Wang et al., 2017) or translocation of high mobility group box-1 (HMGB1) from nuclei (Biran et al., 2017). The colocalization of DNA damage foci (typically assessed by γH2AX immunostaining) and telomeres by ImmunoFISH (telomere-associated foci, TAF) (Herbig et al., 2006, Hewitt et al., 2012) is a powerful indicator for a persistent DDR driving senescence. Importantly, sensitivity/specificity of any one of these senescence biomarkers is insufficient for a decisive identification of a senescent cell. This has multiple causes: i) most of the markers named above (with the possible exception of TAF) are also expressed by cells in stress situations that not necessarily lead to senescence; ii) senescence is a complex phenotype that can vary significantly depending on cell type, senescence inducer and kinetic factors such that not every senescent cell will express all the markers listed, and iii) methodological issues with marker sensitivity that so far, despite some interesting progress (Biran et al., 2017), exclude a fully quantitative assessment of senescent cell frequencies in any tissue. Accordingly, the combined use of multiple biomarkers is now a standard requirement for the identification of changes in senescence abundance (Gorgoulis et al., 2019, von Zglinicki, 2021).

Although senescent cells are unable to divide, they remain metabolically active (de Magalhães and Passos, 2018), which allows them to develop a plethora of phenotypic changes or ‘building blocks’ (von Zglinicki, 2021). These building blocks interact closely with each other, leading to the long-term stability of the senescence stress response. For instance, Senescence-Associated Mitochondrial Dysfunction results in enhanced ROS production that stabilizes senescence by maintaining a persistent DDR (Passos et al., 2010). In fact, mitochondria are essential for establishment of cell senescence (Correia-Melo et al., 2016).

A very important ‘building block’ is the so-called Senescence-Associated Secretory Phenotype (SASP). Senescent cells release a large number of biologically active molecules, including pro-inflammatory cytokines and chemokines, growth factors and matrix metalloproteinases (Coppé et al., 2008) but also non-peptide factors including vesicles encapsulating miRNAs, oxidized lipids, reactive oxygen species and others (Nelson et al., 2012, Weilner et al., 2013). The composition of the SASP varies widely with cell type, senescence inducer, kinetic progression through the senescent phenotype and, probably, a host of additional factors (Kirschner et al., 2020). For instance, fibroblasts that enter senescence initially predominantly secrete anti-inflammatory chemokines and TGF-β in a pathway driven by Notch-1 (Hoare and Narita, 2017), while later ROS- and p38MAPK-induced activation of NF-kB and C/EBPbeta results in a pro-inflammatory SASP (Acosta et al., 2008, Coppé et al., 2008). Recently, COX-2 emerged as another critical regulator of SASP composition (Gonçalves et al., 2021). The SASP attracts phagocytic immune cells and thus contributes to senescence resolution. However, it also stabilizes senescence in an autocrine fashion (Acosta et al., 2008, Kuilman et al., 2008) and can even induce it in neighboring somatic cells (Acosta et al., 2013, Nelson et al., 2012), thus amplifying senescence within tissues (da Silva et al., 2019) and system-wide (Xu et al., 2018b, Xu et al., 2018a). SASP components including TGF-β modulate differentiation and proliferation capabilities of stem and progenitor cells (Xu et al., 2018a, Xu et al., 2018b). SASP interleukins and chemokines contribute to tissue and systemic chronic inflammation (Franceschi et al., 2000, Lee et al., 2021b) and MMPs secreted as part of the SASP may cause matrix remodeling and degeneration (Varela-Eirín et al., 2018).

Accordingly, a recent definition describes senescence as a stress response comprising proliferation arrest together with a range of phenotypic building blocks (SASP, mitochondrial dysfunction, epigenetic reprogramming, etc.) in close interaction (Gorgoulis et al., 2019, von Zglinicki, 2021). This implies that even post-mitotic cells can undergo the same type of stress response. In fact, neurons, myofibers and other postmitotic cells have been found to accumulate senescence-like changes with age (von Zglinicki et al., 2020).

### 2.2 Cell senescence and ageing in vivo

Senescent cells accumulate in many, if not all, organs, and tissues during ageing (Wang et al., 2009) as well as in a wide range of degenerative pathologies including, for instance, atherosclerosis (Erusalimsky and Kurz, 2005), osteoarthritis (Martin and Buckwalter, 2003), chronic lung disease (Noureddine et al., 2011), and precancerous lesions (Collado et al., 2007). The accumulation of senescent hepatocytes and intestinal enterocytes has been found to be a predictor of the physiological age of mice in different ageing models (Jurk et al., 2014). However, evidence for a causal role of senescent cells in ageing, age-related diseases and tumorigenesis came from recent intervention studies that specifically ablated senescent cells in mice (termed ‘senolytics’). In pharmacogenetic approaches, senescent cells were targeted via the p16INK4a promoter, which was used to drive a suicide gene following pharmacological induction (Baker et al., 2016, Baker et al., 2011, Demaria et al., 2014). Furthermore, pharmacologic agents that more or less specifically target cell death pathways in senescent cells were also discovered (Fuhrmann-Stroissnigg et al., 2017, Zhu et al., 2017, Zhu et al., 2016, Zhu et al., 2015), providing tools that can be translated into the clinic. Intervention experiments with such senolytic approaches in mice postponed age-associated functional losses and degradative diseases in a wide range of organ systems, including muscle, liver, lung, bone, the cardiovascular system, and the brain (for review see Robbins et al., 2021). Importantly, senolytic interventions also enhanced median lifespan in mice (Baker et al., 2016, Yousefzadeh et al., 2018). Moreover, transplantation of low numbers of senescent cells into the visceral fat of mice impaired physical function and shortened lifespan of old animals in a dose-dependent manner, and these effects were rescued by senolytic intervention (Xu et al., 2018b, Xu et al., 2018a).

Based on the promising results of pre-clinical experiments in mice, over one dozen FDA-approved clinical trials with the senolytics Dasatinib and Quercetin (D+Q), Fisetin, and UBX0101 have been instigated to test the systemic treatment of multiple age-related conditions and diseases in humans. To date, three trials have reported results. Results of the trial using UBX0101 to treat osteoarthritis have been disappointing to date (https://clinicaltrials.gov/ct2/show/NCT04229225). However, early studies with D+Q appeared more promising. D+Q was used in an open-label phase I pilot study to treat subjects with diabetic kidney disease (https://clinicaltrials.gov/ct2/show/NCT02848131). Results so far strongly suggested that D+Q decreased the burden of senescent cells in humans (Hickson et al., 2019). This trial is continuing to ascertain whether senolytics alleviate diabetes and its complications. An open-label, two-center study of intermittent D+Q treatment in patients with idiopathic pulmonary fibrosis (https://clinicaltrials.gov/ct2/show/NCT02874989) also was conducted and showed improvements in physical function parameters such as gait speed and chair stand time in the treated group (Justice et al., 2019). Clinical trials testing Fisetin for chronic kidney disease (https://clinicaltrials.gov/ct2/show/NCT03325322), skeletal health (https://clinicaltrials.gov/ct2/show/NCT04313634), frailty (https://clinicaltrials.gov/ct2/show/NCT03675724), and osteoarthritis (https://clinicaltrials.gov/ct2/show/NCT04210986) are under way; no results have been reported yet. Finally, two trials testing Fisetin for the prevention of disease progression and death in the elderly infected with SARS-CoV-2 (severe acute respiratory syndrome–coronavirus 2) were recently registered with the National Institutes of Health (https://clinicaltrials.gov/ct2/show/NCT04476953 and https://clinicaltrials.gov/ct2/show/NCT04537299). Taken together, these results strongly suggest that cell senescence is a significant contributor to ageing in many organs and that senolytic agents hold promise for treating major age-related diseases.

However, is this also the case for ageing of the skin?

## 3 Evidence for a role of cell senescence in intrinsic skin ageing

### 3.1 Correlative data: senescent cells accumulate during skin ageing

**Fig. 3:**
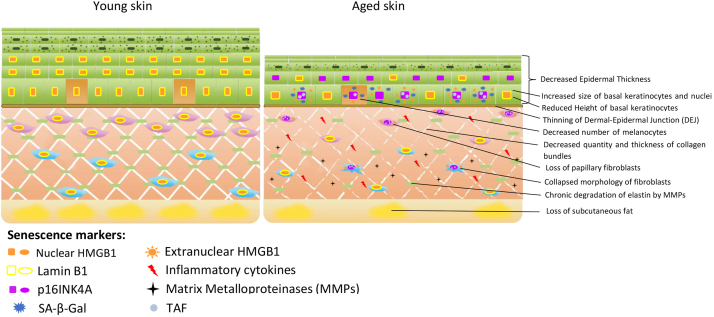
Cell senescence and intrinsic skin ageing. Keratinocytes, melanocytes and fibroblasts express multiple markers of cell senescence associated with physiological indicators of skin ageing, including epidermal thinning and reduced keratinocyte differentiation, decrease of papillary fibroblast frequencies, remodeling of the dermal extracellular matrix and loss of subcutaneous fat. See text for further discussion.

There is good evidence that senescent cells accumulate in essentially all compartments of the skin during ageing, not only in sun-exposed skin (Cavinato and Jansen-Dürr, 2017, Fitsiou et al., 2021) but also during intrinsic ageing of sun-protected skin (see Fig. 3 and Table 2). A recent systematic review finds a significant association between senescent cell abundance in skin and donor age (Tuttle et al., 2020). However, the present knowledge about senescence accumulation during ageing in important cell types in the skin is incomplete and, for some cell types, non-existent (Table 2).

**Table 2:** Overview of skin cell senescence.

localization	Cell type	Senescence markers	Main references
Epidermis	keratinocytes	SA-β-Gal MMP-1, MMP-3, MMP-9 LaminB1 P21WAF1/CIP1, TAF, HMGB1 p16INK4a HMGB1	(Dimri et al., 1995) (Velarde et al., 2012) (Quan et al., 2009) (Wang et al., 2017) (Victorelli et al., 2019) (Ressler et al., 2006) (Waaijer et al., 2012) (Johnson et al., 2013)
Str basale	melanocyte	p16INK4a, TAF, HMGB1 LaminB1 (nevi) IL-6 (nevi)	(Victorelli et al., 2019) (Ivanov et al., 2013) (Ghosh and Capell, 2016)
Epidermis	Langerhans cells	unknown	(Lee, 2021, Lee et al., 2021a, Lee et al., 2021b)
Dermis	Fibroblasts	TAF, p16INK4a p16INK4a Nuclear morphology	(Herbig et al., 2006) (Ressler et al., 2006) (Waaijer et al., 2012)
Dermal vasculature	Endothelial cells	In skin: unknown Other tissues: p16, p21WAF1/CIP1, Sen-b-Gal, SASP	(Sun and Feinberg, 2021)
Dermal vasculature	Smooth muscle cells	In skin: unknown Other tissues: p16, p21WAF1/CIP1, Sen-b-Gal, SASP	(Chi et al., 2019)
Dermis	macrophages	In skin: unknown Other tissues: p16, Sen-β-Gal in response to stimulation (non-senescent)	(Guimarães et al., 2021) (De Maeyer and Chambers, 2021) (Hall et al., 2017)
Dermis	Dermal dendritic cells	Unknown	
Dermis	Mast cells	Unknown	
Dermis	γδ T cells	Unknown	

#### 3.1.1 Epidermal keratinocytes

The epidermis is made up of a highly proliferative stratified squamous epithelium (Fig. 1). Stem cells in the basal keratinocyte layer express telomerase (Härle-Bachor and Boukamp, 1996, Krunic et al., 2009) and can thus retain their telomere length, at least during ageing of UV-protected skin (Krunic et al., 2009, Victorelli et al., 2019). Stem cells produce transient amplifying progenitor cells that detach from the basement membrane (Rangel-Huerta and Maldonado, 2017). Their progeny migrates towards the epithelial surface and terminally differentiates towards corneocytes, the whole process taking place over 3–6 weeks. Therefore, the epidermis can eliminate extrinsic macromolecular damage through the desquamation of terminally differentiated keratinocytes, thus precluding damage accumulation and rendering the tissue compartment comparatively stress resistant. Under high levels of oxidative stress, e.g., induced by UVB, keratinocytes, in contrast to fibroblasts, can undergo apoptosis as a DNA damage response (Van Laethem et al., 2005). These findings led to the view that keratinocytes might show very little senescence accumulation during ageing (Ho and Dreesen, 2021). However, in the intestine, an epithelium with even faster turnover, frequencies of senescent cells specifically in the progenitor compartment increase significantly during ageing, and this was driven by telomere uncapping without shortening (Hewitt et al., 2012, Jurk et al., 2014, Wang et al., 2009). Similarly, in human epidermis, specifically the stratum basale and spinosum/granulosum, cells bearing senescence markers including Sen-β-Gal (Dimri et al., 1995, Velarde et al., 2012), p16INK4A (Ressler et al., 2006, Rübe et al., 2021, Waaijer et al., 2016, Waaijer et al., 2012), p21WAF1/CIP1 (Victorelli et al., 2019), TAF (Victorelli et al., 2019), H2A.J (Rübe et al., 2021), loss of Lamin B1 (Dreesen et al., 2013, Wang et al., 2017) and of HMGB1 (Victorelli et al., 2019) have been observed to accumulate with (biological) age. Unfortunately, only few studies did clearly discriminate between keratinocytes and melanocytes as the next frequent cell type in the epidermis. When this was done, it was found that in the epidermis from old donors, almost all cells expressing high levels of p16INK4A were melanocytes (Victorelli et al., 2019, Waaijer et al., 2016). This result has been interpreted to suggest that i) melanocytes present the main population of senescent cells in the epidermis, and ii) that p16INK4A might not qualify as a marker of senescence in keratinocytes. We will discuss notion i) further below (chapter 3.1.2) but would like to caution against notion ii) here because high p16INK4A has been shown consistently in primary human keratinocytes in replicative, oncogene- und stress-induced senescence (Kim et al., 2015, Miyake et al., 2019, Sasaki et al., 2014, Tinaburri et al., 2018), and in primary mouse keratinocytes in oncogene- and stress-induced senescence (Ritschka et al., 2017) and inactivation of p16INK4A immortalized primary human keratinocytes (Maurelli et al., 2006). There are also arguments against the idea that p16INK4A could be upregulated in keratinocytes only during in-vitro, but not during in-vivo senescence: p16INK4A is enhanced in primary keratinocyte cultures from old donors (Tinaburri et al., 2018) and when skin was reconstituted from keratinocytes and fibroblasts only, high p16INK4A was found in the basal layer when the reconstitution was done with keratinocytes from old donors (Adamus et al., 2014). Interestingly, it has been suggested that melanocytes compared to keratinocytes present much higher levels of p16INK4A because this acts to repress high ROS levels that are a by-product of melanin generation irrespective from cell cycle regulation (Jenkins and Grossman, 2013, Jenkins et al., 2011). It seems a possibility that the p16INK4A levels in senescent keratinocytes in situ might well be below the level of melanocyte p16INK4A and thus below the detection threshold in comparative immunostaining.

#### 3.1.2 Melanocytes

Melanocytes are located at the *stratum basale* of the epidermis. They comprise approximately 1–2% of epidermal cells and are thus the second most numerous epidermal cell type after keratinocytes. They produce photoprotective melanin pigments, which are transferred to adjacent keratinocytes. Differentiated melanocytes have extremely low proliferative capacity (Taylor et al., 2011), which might suggest that they are unlikely to undergo replicative senescence. However, melanocytes have long been thought to readily undergo telomere-dependent (but apparently not p21WAF1/CIP1-dependent) senescence (Bandyopadhyay and Medrano, 2000), and this has been recognized as an important mechanism to suppress melanoma development (Suram et al., 2012). Accordingly, melanocytes expressing senescence markers including high p16INK4a were consistently observed in aged human skin and associated with hyperpigmentation and other morphological markers of biological age (Victorelli et al., 2019, Waaijer et al., 2016). Moreover, evidence for melanocyte-specific telomere dysfunction without significant telomere shortening (indicated by TAF) was observed in skin from older donors (Victorelli et al., 2019), suggesting that melanocytes are susceptible to undergo telomere length-independent stress-induced senescence (Victorelli and Passos, 2017).

The suggestion that melanocytes might constitute the major senescent cell population in the epidermis was based on the almost exclusive co-localization of high p16INK4A and melanocyte markers S-100 and Melan-A in aged epidermis (Victorelli et al., 2019, Waaijer et al., 2012). It was further supported by showing that senescent keratinocytes clustered around senescent melanocytes and that senescent melanocytes can induce senescence in neighboring cells via paracrine signaling involving IP10, CXCR3 and ROS (Victorelli et al., 2019). However, as discussed above, high p16INK4A levels in melanocytes might be partially independent of senescence. Moreover, paracrine interactions between senescent and non-senescent skin cells are most probably more complex: (Victorelli et al., 2019) showed that senescent melanocytes can induce senescence in keratinocytes in 3D epidermal models, but the reverse situation, e.g. the induction of melanocyte senescence from senescent keratinocytes or fibroblasts has not been formally examined. However, it has been shown that senescent fibroblasts can induce skin hyperpigmentation, which in turn might drive melanocyte senescence (Yoon et al., 2018). Furthermore, stress-induced activation of p53 in keratinocytes drives paracrine induction of melanogenesis (Murase et al., 2009), see also (Lee, 2021, Lee et al., 2021a, Lee et al., 2021b) for review), which is associated with enhanced ROS production that might cause melanocyte senescence. At the present state of knowledge, it seems to be safe to assume that senescence in the epidermis (and possible in the complete skin) can arise in any of the main cell types and can be transmitted via paracrine bystander signaling to any other cell in the neighborhood.

#### 3.1.3 Dermal fibroblasts

Unlike the epidermis, the dermis is unable to remove extrinsic damage by cell shedding, and fibroblasts, the main cell type in the dermal matrix, proliferate very slowly in vivo (Tigges et al., 2014). The loss of protein homeostasis in aged fibroblasts can led to accumulation of misfolded and damaged proteins (Tigges et al., 2014), which may cause them to senesce (Rodier et al., 2009). Fibroblasts might be more prone to accumulation of DNA damage (and thus senescence) than keratinocytes, because they are less efficient in global nucleotide excision repair and have lower antioxidant capacities (D'Errico et al., 2007). Accordingly, the accumulation of fibroblasts bearing multiple senescence markers in ageing skin has been documented in mice, baboons and humans (Dimri et al., 1995, Herbig et al., 2006, Lewis et al., 2011, Ressler et al., 2006, Waaijer et al., 2012, Wang et al., 2009). However, quantitative estimates of senescent cell frequencies varied widely among studies. For instance, in mouse dermis, frequencies of γH2AX-positive fibroblasts increased from about 1% in adults to 5% in animals aged 42 months (Wang et al., 2009). In the dermis of old (>25 years) baboons, about 20% of the fibroblasts were TAF-positive and up to 80% were positive for the heterochromatin marker HIRA (Herbig et al., 2006). In human dermis, the numbers of p16-positive cells increased at least two-fold between middle and old age (below and above 70 years) (Ressler et al., 2006). Using nuclear morphology as a marker of senescence, (Lewis et al., 2011) estimated an increase of senescent cells from about 25 to almost 60% in sun-protected papillary dermis in a cross-sectional comparison of young adult and geriatric volunteers. One important source of the variation between studies is the use of different senescence markers which are often unvalidated or not sensitive. Therefore, it still remains unclear how many senescent cells accumulate in aged skin and to which extent species or anatomical localization differences might have an impact on the rate of senescent cell accumulation.

An insufficiently examined question relates to the differences in ageing between papillary and reticular fibroblasts and the role of cell senescence in it. Fibroblasts in the papillary region of the dermis in young donors are smaller, have higher growth rates, secrete fewer cytokines and matrix metalloproteinases and support keratinocyte differentiation better than reticular fibroblasts. However, during ageing fibroblasts in the papillary compartment become more similar to reticular ones, while properties of the reticular population remain unchanged (Janson et al., 2017, Lämmermann et al., 2018, Mine et al., 2008). These results may be interpreted differently. For instance, papillary and reticular fibroblasts might belong to different lineages, and papillary fibroblasts might (trans-)differentiate into reticular ones during ageing (Korosec et al., 2019, Lämmermann et al., 2018). Another possibility could be that reticular fibroblasts from young donors already contain a higher fraction of senescent cells, and that papillary fibroblasts reach similar senescent fractions only in old skin. This interpretation is in accordance with data showing that treatment of reticular fibroblasts with a senolytic drug changed their phenotype towards papillary cells, while it had no effect on papillary fibroblasts from a young donor. Multiple senescence markers need to be analyzed separately on papillary and reticular fibroblasts to address this question.

#### 3.1.4 Other cell types in the skin

In subcutaneous fat, adipocyte progenitors become senescent with increasing donor age (Sepe et al., 2011, Tchkonia et al., 2010). Frequencies of senescent preadipocytes in subcutaneous fat (assessed as γH2AX-positive, Ki67-negative cells) increased from about 3% to 10% with age in both humans and rats (Xu et al., 2015). Presence of senescent fat cells in the subcutaneous layer has been associated with chronic inflammation (Palmer et al., 2019), however, whether subcutaneous senescent preadipocytes can enhance senescence in other layers of the skin has not been examined yet.

It is well established that skin-resident immune cells respond to the chronic low-level inflammation associated with skin ageing (Lee, 2021, Lee et al., 2021a, Lee et al., 2021b). However, to our knowledge, no information is available about whether these cells senesce and to what extent this may contribute to skin ageing. Similarly, there is essentially no information available about senescence in the dermal vasculature (Tab 2).

### 3.2 Modifying frequencies of senescent cells in 3D skin equivalents

Tissue engineered organotypic models are valuable tools to study the influence of senescent cells in human skin ageing. Bioengineered skin equivalents capture the multicellular and multi-layered complexity of human skin in vitro and enable interactions between different cell populations to be studied. To study the contribution of senescence to the ageing phenotype in vitro, 3D skin-equivalents have been developed that incorporate senescent cells (Diekmann et al., 2016, Janson et al., 2013, Lämmermann et al., 2018, Weinmüllner et al., 2020). A major limitation of these models is their simplified composition: frequently they consist of matrix-embedded fibroblasts and keratinocytes only. While epidermal models composed of keratinocytes and melanocytes have been successfully used for ageing studies (Victorelli et al., 2019), effects of ageing/senescence in skin 3D equivalents containing resident immune cells have not yet been studied to our knowledge. Moreover, the interactions of different dermal equivalent matrices with senescent skin cells have not been systematically examined.

In a study which incorporated mitomycin C-induced senescent fibroblasts into 3D skin equivalents, age-related changes including an increase in matrix-degrading enzymes, the prominent degradation of the extracellular matrix networks, and decreased filaggrin expression in the upper epidermis were observed (Diekmann et al., 2016). Another study developed human skin equivalents using early or late passage fibroblasts, to mimic young and aged skin. The incorporation of late passage fibroblasts produced a thinner dermis, which may be explained by an increase in MMP1 secretion. These models also provided evidence of dermal-epidermal crosstalk, as the late passage fibroblasts induced alterations in epidermal differentiation such as an increased expression of keratin 6 and a decrease in keratin 10 (Janson et al., 2013). A study focusing on the impact of epidermal-dermal crosstalk with age found that increasing the ratios of either oxidative stress-induced or doxorubicin-induced senescent cells within the dermis caused a progressive thinning of the epidermis. In addition to epidermal thinning, the co-culture with senescent fibroblasts also altered epidermal differentiation and barrier function in the model, with a decrease in keratin 10 and filaggrin, and partial impairment of barrier integrity as measured by biotin permeability assay (Weinmüllner et al., 2020). Conversely, treatment of full thickness 3D skin models with an alcoholic extract of *Solidago alpestris*, which had weak senolytic activity in vitro and suppressed the SASP, enhanced the papillary phenotype of fibroblasts in the dermal layer and improved epidermal differentiation (Lämmermann et al., 2018).

Keratinocyte senescence had similar effects in 3D organotypic cultures: Increasing the expression of p16INK4a in keratinocytes from young donors yielded a thinner epidermal layer like that from older donors, while decreasing p16INK4A in keratinocytes from older donors resulted in improved epidermal thickness (Adamus et al., 2014). Similarly, specific ablation of senescent cells in human epidermal equivalents containing UV-induced senescent melanocytes by treatment with the senolytic drug ABT-737 reduced frequencies of senescent melanocytes and increased the thickness of the epidermal equivalents. Similar results were obtained by treatment with the mitochondria-targeted antioxidant MitoQ, in agreement with a causal role for oxidative stress in UV-induced senescence of melanocytes (Victorelli et al., 2019).

### 3.3 In vivo senolytic and senostatic interventions: effects on skin ageing

Until now, there are few studies that report the impact of a modification of senescent cell frequencies on skin ageing in vivo. A summary of these is given in Table 3.

**Table 3:** Effects of senolytic and senostatic interventions on skin ageing.

Anti-senescence intervention	Effects on skin ageing	Species
ABT-737 (senolytic)	Increased hair follicle stem cell proliferation, reduced senescent melanocytes, rescued epidermal atrophy (Yosef et al., 2016) Reduction of compensatory hyperplasia and papilloma inducing WNT activity in mice overexpressing p16INK4a in basal keratinocytes (Azazmeh et al., 2020)	Mouse Mouse
D+Q (senolytic)	Reduction of p16INK4a and p21WAF1/CIP1-positive epidermal cell frequencies (Hickson et al., 2019)	Human
Fisetin (senolytic)	Topical application to UV-irradiated skin improved TEWL and inflammation markers (Wu et al., 2017)	Mouse
Thermocoagulation in upper dermis	Reduced skin pigmentation and increased lightness in senile lentigo (Yoon et al., 2018)	Human
Dermabrasion	Improved collagen synthesis and UV response in sun-protected aged skin (Lewis et al., 2011)	Human
Rapamycin	Topical application reduced wrinkles and sagging of skin, increased dermal volume, increased expression of collagen VII (Chung et al., 2019)	Human
Metformin	Reduced UVB-induced epidermal hyperplasia and carcinogenesis (Wu et al., 2013)	Mouse

#### 3.3.1 Murine models

A few studies have experimentally modified numbers of senescent cells in mouse skin. Deletion of the antioxidant gene SOD2 in mice resulted in increased oxidative stress, mitochondrial dysfunction and induction of DNA damage in skin. Concomitantly, increased SA-β-Gal activity in the epidermis and higher p16INK4a levels in skin were observed, together with increased transglutaminase1 and decreased keratin K10 and K1 mRNA levels and decreased epidermal thickness, indicating enhanced terminal differentiation in the epidermis of mice with high oxidative stress and increased senescent cell frequencies (Velarde et al., 2012). The increase of senescent, p16INK4A-positive cells might be responsible for changes in connective tissue abundance and composition in this model (Treiber et al., 2011). Induction of senescence in basal keratinocytes by induced expression of p14ARF under control of the keratin K5 promoter resulted in persistently enhanced numbers of basal keratinocytes displaying Sen-β-Gal positivity and other markers of senescence. This resulted in hair follicle stem cell dysfunction evident by a dramatic decrease in the numbers of developed hair follicles, and progressive and persistent hair loss (alopecia). Effects on epidermal thickness were inconsistent, because of a transient induction of hyperproliferation from p14ARF-nonexpressing stem cells (Tokarsky-Amiel et al., 2013). Similarly, increased numbers of senescent basal keratinocytes, stem cell dysfunction and alopecia were seen following overexpression of a K5-p16INK4a transgene (Azazmeh et al., 2020). However, long-term activation of the transgene (for 6 months) did not resemble a premature ageing phenotype in the epidermis. Rather, it resulted in epidermal hyperplasia and dysplasia with hyperproliferation of (mostly) p16INK4a-nonexpressing basal cells, but delayed regeneration after wounding. Hyperplasia and formation of epidermal papilloma after carcinogen treatment was induced via WNT signaling from p16INK4a-expressing, partially senescent cells.

The Bcl-2 protein-family inhibitor ABT-737 is a first-generation senolytic, and treatment of K5-p16INK4a transgenic mice after 6 months of induction of the transgene reduced p16INK4a-positive cells, reversed hyperplasia and diminished activation of the WNT pathway (Azazmeh et al., 2020). Similarly, treatment of K5-p14ARF mice (Tokarsky-Amiel et al., 2013) with ABT-737 efficiently eliminated senescent cells from the epidermis leading to an increase in hair-follicle stem cell proliferation (Yosef et al., 2016).

Fisetin is a senolytic with a specificity for endothelial cells (but not for fibroblasts or adipocytes) (Zhu et al., 2017) that has been shown to increase health span in mice (Yousefzadeh et al., 2018). When applied daily for 10 weeks to skin of UV-irradiated hairless mice it improved TEWL and reduced inflammation (Xiao et al., 2021).

Caloric restriction (da Silva et al., 2019, Ogrodnik et al., 2017) and caloric restriction mimetics like rapamycin (Blagosklonny, 2018) or metformin (Moiseeva et al., 2013) inhibit the senescent phenotype, specifically the SASP, at least partially by suppressing signaling through mTORC1 and mTORC2, which is persistently activated in senescence (Carroll et al., 2017). The senostatic activity of these interventions suppresses the spreading of the senescent phenotype from one cell to the other, helping to reduce senescent cell numbers in immunocompetent hosts (da Silva et al., 2019) and making the mTOR pathway a target for senescent cell elimination. Rapamycin efficiently slowed down ageing in mice (Harrison et al., 2009, Wilkinson et al., 2012) but effects on skin ageing were not reported in these studies. There is little evidence for an age-delaying effect of caloric restriction specifically in skin (Choi, 2020). Topical application of rapamycin did not improve cutaneous wound healing, although metformin had a strong positive effect (Zhao et al., 2017). Metformin reduced the SASP of UV-irradiated keratinocytes in vitro and alleviated UVB-induced skin damage in mice after topical administration (Xiao et al., 2021).

#### 3.3.2 Clinical studies

A study with 9 patients with diabetic kidney disease recently showed that a short systemic intervention with the senolytic combination of Dasatinib and Quercetin can reduce frequencies of senescent cells (assessed as p16INK4a- and p21WAF1/CIP1-positive cells) not only in adipose tissue but also in the epidermis, together with a reduction in circulating SASP factors. However, effects on skin function or quality were not reported (Hickson et al., 2019).

Interventions aimed at improving aged skin function in humans so far relied on more indirect measures to reduce skin cell senescence. Dermabrasion is known to promote collagen remodeling and re-epithelialisation (Smith, 2014). In a small study with geriatric volunteers, it reduced frequencies of senescent fibroblasts in the upper dermis, increased papillary thickness, upregulated IGF1 expression and improved the UVB response in sun-protected aged skin (Lewis et al., 2011). Senescent fibroblasts in the upper dermis were shown to enhance melanin production and drive skin hyperpigmentation in vitro and *ex vivo*. Accordingly, 10 volunteers with senile lentigo were treated with microneedle radiofrequency for 6 weeks, which reduced both frequencies of p16INK4a-positive cells in the upper dermis and epidermal pigmentation in the treated area (Yoon et al., 2018).

Metformin has frequently been used for treatment of cutaneous disorders including skin tumors, psoriasis, hyperpigmentation (Bubna, 2016). Whether any of the beneficial effects are related to its senostatic properties, has not been assessed. Topical rapamycin has been applied in multiple clinical studies, mostly for treatment of patients with angiofibromas linked to tuberous sclerosis complex (Leducq et al., 2019). More recently, a small randomized prospective clinical trial was performed to test whether topical application of rapamycin in low concentration could reduce senescence markers and improve function and appearance of photoaged skin (ClinicalTrials.gov Identifier: NCT03103893). Topical rapamycin reduced the expression of p16INK4a consistent with a reduction in cellular senescence. This change was accompanied by an improvement in clinical appearance of the skin and histological markers of aging and by an increase in collagen VII, which is critical to the integrity of the basement membrane (Chung et al., 2019).

Finally, it is interesting to note that the hereditary premature ageing disorders Werner syndrome, Xeroderma pigmentosum and Hutchinson-Gilford progeria are all due to defects in DNA repair or nuclear organization and thus associated with accelerated cellular senescence and greatly enhanced age-related phenotypes in the skin (Davis et al., 2007, Demaria et al., 2015, Liu et al., 2006).

In conclusion, there is a good amount of pre-clinical and clinical data showing a strong positive correlation between reduction of senescent cells frequencies and functional improvement of skin.

## 4 Conclusions and open questions

Research in recent years has convincingly proven that cell senescence is a pathophysiologically relevant cause of age-associated multimorbidity and functional losses. Organism-wide ablation of senescent cells has resulted in prolonged lifespan and better maintenance of function in multiple organs and tissues (Robbins et al., 2021), while senescent cell transplantation caused shortened lifespan and ageing-associated dysfunction in remote organs (Xu et al., 2018b, Xu et al., 2018a). Via a generalized SASP, senescence spreads from cell to cell in vitro (Nelson et al., 2012) and in vivo (Acosta et al., 2013, da Silva et al., 2019, Xu et al., 2018a). As tools to ablate senescent cells specifically in one cell type or organ are only now emerging, discrimination between systemic and cell-autonomous effects of senescence has only seldom been achieved; the identification of a cell-autonomous induction of steatosis in senescent hepatocytes being an exception (Ogrodnik et al., 2017).

Whether senescence of skin cells makes a significant causal contribution to skin ageing can still not be conclusively decided. Senescent cells accumulate with age in all major compartments of the skin, and the inclusion of increased numbers of senescent cells recapitulates at least some ageing-associated changes in human 3D skin models (see chapters 3.1 and 3.2 above). However, animal intervention experiments and clinical intervention trials have been less conclusive about the role of senescence in skin ageing as compared to other organs (see chapter 3.3) for several different reasons:

i) Studies with transgenic induction of senescence in keratinocytes (Azazmeh et al., 2020, Tokarsky-Amiel et al., 2013) recapitulated some features of intrinsic skin ageing (e.g., loss of regenerative capacity), but also revealed phenotypes not normally seen during ageing (for instance, compensatory hyperproliferation). This might however not be surprising as skin is a highly regenerative organ, requiring an exact long-term balance of proliferative and differentiation activities of stem and progenitor cells. Senescent cells can significantly impact this balance via a multitude of factors secreted as part of the SASP. Importantly, SASP factors can either stimulate (Antelo-Iglesias et al., 2021) or inhibit (Ogrodnik et al., 2019) regeneration. For instance, transient senescence was found to be a requirement for successful skin wound healing (Demaria et al., 2014, Jun and Lau, 2010), while correlative data indicate a role for persistent, pre-existing senescence for chronicity of wounds in old and/or diabetic individuals (Stanley and Osler, 2001). Given how little is known how different factors including cell type, senescence inducer or progression of the phenotype determine SASP composition (Kirschner et al., 2020), it will be very complicated to predict how senescent cells will change proliferation and differentiation in the epidermis.
ii) Successful intervention studies have often been performed with rather unspecific interventions. Caloric restriction or metformin, for example, have anti-senescence activities, but in addition act via a plethora of molecular pathways, some of which may be related to senescence but others not (Hu et al., 2021). Similarly, increasing oxidative stress in skin e.g., (Velarde et al., 2012) might or might not primarily impact via senescent cell generation.
iii) While the reduction of senescence markers in skin after systemic treatment with the senolytic combination Dasatinib + Quercetin has been demonstrated in one clinical study (Hickson et al., 2019), clinical studies showing improvement of skin function after senolytic intervention are still missing. Similarly, we did find only a single preclinical study showing relief of UV damage in mouse skin following topical senolytic (fisetin) application (Wu et al., 2017).
iv) These limitations in the present knowledge clearly indicate some factors that require more experimental consideration by the research community. Evidently, it will be necessary to see whether, and to which extent, specific ablation of senescent cells (and ideally, of specific cell types in the skin) can slow down skin ageing in vivo. Importantly, it needs to be tested whether ablation of senescent cells in aged skin before wounding can improve the healing of wounds that otherwise would become chronic. It is necessary to understand more about the differences between persistent and transient senescent cells in skin. Are they experiencing intrinsically different senescence phenotypes, or is a difference in senescence kinetics more important? Also, very little is known about how the composition of the extracellular matrix in the dermal layer, which changes significantly with age (McCabe et al., 2020), changes properties of senescent cells. It is interesting to note that the inclusion of fibroblasts that were rendered senescent in 2D culture into a collagen matrix resulted in reduced proliferation and differentiation within the epidermal layer in 3D full thickness skin equivalents (Weinmüllner et al., 2020), while the same senescent fibroblasts when embedded in a polystyrene scaffold caused a thickening of the epidermal layer (unpublished). Whether these differences might be informative with respect to the different mechanisms determining intrinsic vs UV-induced skin ageing, remains to be seen. Finally, there is insufficient understanding of how different cell types interact in the skin during progression to senescence. Are melanocytes the primary cell type that becomes most easily senescent in skin as suggested (Victorelli et al., 2019, Waaijer et al., 2016), or are they just more sensitive to secondary signals from senescent keratinocytes or fibroblasts?

Taken together, we feel that the evidence that cellular senescence is a relevant cause of skin ageing is highly suggestive but needs improvement to become conclusive. However, there is strong evidence existing today to assume that better understanding of cell senescence in skin may lead to a breakthrough in interventions into skin ageing.

## Funding

Work leading to this paper was funded by BBSRC-Proctor&Gamble IPA grants BB/S006710/1 (TvZ) and BB/S007431/1 (SP) and by H2020 WIDESPREAD grant 857524 (TvZ and SM). The sponsors had no involvement in study design; in the collection, analysis and interpretation of data; in the writing of the report; and in the decision to submit the article for publication.
